# Effects of Chronic Ascariasis and Trichuriasis on Cytokine Production and Gene Expression in Human Blood: A Cross-Sectional Study

**DOI:** 10.1371/journal.pntd.0001157

**Published:** 2011-06-07

**Authors:** Miguel Reina Ortiz, Fernanda Schreiber, Susana Benitez, Nely Broncano, Martha E. Chico, Maritza Vaca, Neal Alexander, David J. Lewis, Gordon Dougan, Philip J. Cooper

**Affiliations:** 1 Centro de Investigaciones, Fundación Ecuatoriana Para Investigación en Salud, Quinindé, Ecuador; 2 Colegio de Ciencias de la Salud, Universidad San Francisco de Quito, Quito, Ecuador; 3 Wellcome Trust Genome Campus, Wellcome Trust Sanger Institute, Hinxton, United Kingdom; 4 Infectious Disease Epidemiology Unit, London School of Hygiene and Tropical Medicine, London, United Kingdom; 5 St. George's Vaccine Institute, St. George's University of London, London, United Kingdom; 6 Centre for Infection, St. George's University of London, London, United Kingdom; 7 Molecular and Biochemical Parasitology, Liverpool School of Tropical Medicine, Liverpool, United Kingdom; University of Washington, United States of America

## Abstract

**Background:**

Chronic soil-transmitted helminth (STH) infections are associated with effects on systemic immune responses that could be caused by alterations in immune homeostasis. To investigate this, we measured the impact in children of STH infections on cytokine responses and gene expression in unstimulated blood.

**Methodology/Principal Findings:**

Sixty children were classified as having chronic, light, or no STH infections. Peripheral blood mononuclear cells were cultured in medium for 5 days to measure cytokine accumulation. RNA was isolated from peripheral blood and gene expression analysed using microarrays. Different infection groups were compared for the purpose of analysis: STH infection (combined chronic and light vs. uninfected groups) and chronic STH infection (chronic vs. combined light and uninfected groups). The chronic STH infection effect was associated with elevated production of GM-CSF (P = 0.007), IL-2 (P = 0.03), IL-5 (P = 0.01), and IL-10 (P = 0.01). Data reduction suggested that chronic infections were primarily associated with an immune phenotype characterized by elevated IL-5 and IL-10, typical of a modified Th2-like response. Chronic STH infections were associated with the up-regulation of genes associated with immune homeostasis (IDO, P = 0.03; CCL23, P = 0.008, HRK, P = 0.005), down-regulation of microRNA hsa-let-7d (P = 0.01) and differential regulation of several genes associated with granulocyte-mediated inflammation (IL-8, down-regulated, P = 0.0002; RNASE2, up-regulated, P = 0.009; RNASE3, up-regulated, p = 0.03).

**Conclusions/Significance:**

Chronic STH infections were associated with a cytokine response indicative of a modified Th2 response. There was evidence that STH infections were associated with a pattern of gene expression suggestive of the induction of homeostatic mechanisms, the differential expression of several inflammatory genes and the down-regulation of microRNA has-let-7d. Effects on immune homeostasis and the development of a modified Th2 immune response during chronic STH infections could explain the systemic immunologic effects that have been associated with these infections such as impaired immune responses to vaccines and the suppression of inflammatory diseases.

## Author Summary

Soil-transmitted helminth (STH or intestinal worm) infections are extremely common infectious diseases of childhood in developing countries. Infections tend be chronic and may last for many years. Chronic STH infections are associated with modulation of the immune response, a consequence of which may be a reduced prevalence of allergic inflammatory diseases such as asthma. The mechanisms by which STH infections suppress inflammatory responses are poorly understood. In this study, we hypothesized that STH infections may affect immune responses through alterations of immune homeostasis (or the steady-state adjustments of the immune system that maintain equilibrium). We investigated the capacity of blood from children classified as having no, light, or chronic STH infections to produce cytokines at rest (i.e. no immunologic stimulation) and the expression of genes in blood samples. Our data show that blood cells of children with chronic STH infections have an altered immune response that is likely to be associated with less allergic inflammation (the modified Th2 response) and that the expression of some inflammatory genes are reduced. Our findings provide insights into the mechanisms by which STH infections suppress immune responses in children to ensure the survival of the parasite and reduce inflammation.

## Introduction

An estimated two billion people worldwide are infected with soil-transmitted helminth (STH or intestinal helminth or geohelminth) parasites of which *Ascaris lumbricoides* and *Trichuris trichiura* are the most prevalent [1]. STH infections are associated with significant morbidity largely related to the nutritional effects of a chronically infected state among pre-school and school age children [2]. STH infections have been associated with impaired immunity to vaccines [3], [4] and mucosal pathogens [5], and the modulation of inflammatory diseases such as asthma [6].

The mechanisms by which chronic human STH infections modulate immune responses are poorly understood. For STH parasites to have modulatory effects at distal sites (e.g. lung) to non-parasite antigens (e.g. aeroallergens), they would be expected to influence systemic immunity. Such influences could be mediated by alterations in immune homeostatic mechanisms that may include the enhanced production of immune modulatory cytokines. Alternatively, alterations in immune homeostasis could occur through effects on mRNA and/or microRNA expression. MicroRNAs (miRNAs) are small non-coding RNAs that play an important role in the post-transcriptional regulation of gene expression and are considered to be critical for the regulation of innate and adaptive immunity [7].

In the present study we investigated the impact of chronic STH infections on immune homeostasis by measurement of the production of a range of cytokines and chemokines by unstimulated peripheral blood mononuclear cells and on mRNA and microRNA expression in unstimulated peripheral blood from children.

## Materials and Methods

### Study population, recruitment, and sampling

The study design was cross-sectional. Children aged 7–12 years attending 6 schools that served rural communities in the District of Eloy Alfaro in Esmeraldas Province in Ecuador, were eligible for inclusion. Schools were visited and a general assembly of parents and children was called in which the study was explained. Parents wishing to enrol their children into the study then underwent a process of informed written consent individually with study investigators. Minor assent was obtained from the children. Parental consent and minor assent was obtained for 676 children (95% of those eligible) aged 7–12 years. Children were asked to provide a total of 4 serial stool samples and a blood sample was collected into tubes containing sodium heparin as anticoagulant (Vacutainer, BD Diagnostics) for measurement of anti-*A. lumbricoides* IgG (measure of exposure to STHs) and IgG4 (measure of chronic infections with STHs) antibodies. Stool samples were examined by two highly experienced laboratory technicians (SB and NB) using the modified Kato-Katz and formol-ethyl concentration techniques [8]. Children were selected into 3 groups: 1) *uninfected* (n = 41) - absence of STH infections in all 4 stool samples and the absence of anti-*A. lumbricoides* IgG and IgG4 antibodies; 2) light infections (n = 45) – presence of STHs in at least one of 4 stool samples, presence of anti-*A. lumbricoides* IgG but no anti-*A. lumbricoides* IgG4 antibodies; and 3) *chronic* infections (n = 61) – presence of STHs (both *A. lumbricoides* and *T. trichiura*) in all 4 stool samples and presence of anti-*A. lumbricoides* IgG and IgG4 antibodies. Children who did not provide all stool samples or did not satisfy the study group criteria with respect to the presence of STH infections and the presence or absence of anti-*A. lumbricoides* IgG and IgG were excluded. A second blood sample was obtained from the 147 children selected into the 3 study groups. Blood samples were collected into Paxgene tubes (PreAnalitiX GmbH, BD Qiagen) for gene expression and into tubes containing sodium heparin as anticoagulant (Vacutainer, BD Diagnostics) for cell culture. Blood samples for culture were analyzed within 5 hours of collection. Paxgene tubes were maintained at ambient temperature in a polystyrene box for 4 hours, then transferred to a −20°C freezer for 24 hours and stored at −80°C before shipping to the Wellcome Trust Sanger Institute on dry ice for analysis. The final selection of 60 children (20 in each infection group) for analysis, a sample size restricted by cost considerations, was based on RNA quantity (>50 ng/µl) and integrity (absence of degradation using the electrophoretic profile provided by Bioanalyzer) after extraction. Sample collection was performed between May and October 2008. The parents of each child received written results for all stool (parasites) and blood samples (blood count and anemia) and treatment where appropriate was provided by the study physician (MR). The ethics committee of the Universidad San Francisco de Quito approved the study protocol.

### Antibody measurements

Parasite-specific IgE and polyclonal IgE antibody levels were analyzed using the UNI-CAP assay (Pharmacia Biotech, Uppsala, Sweden). Anti-*A. lumbricoides* IgG and IgG4 antibodies were measured as described [9]. Positive values were defined as >3 SD above mean values of a pool of 10 uninfected non-endemic control sera. Control sera were collected from health professionals living in the town of Quinindé, Esmeraldas Province. All had four negative stool samples for STH parasites after examination using the modified Kato-Katz and formol-ethyl concentration techniques [8].

### Peripheral blood mononuclear cell (PBMC) cultures

PBMCs were isolated by density gradient centrifugation over Histopaque (Sigma). Cells were cultured at a concentration of 1×10^6^ cells/well in 48-well tissue culture plates in duplicate (Greiner-Labortechnik) with supplemented RPMI 1640 medium (i.e. no immunologic stimulation). Plates were incubated at 37°C and 5% CO_2_ for 5 days. Supernatant fluids were collected at 5 days, stored at −70°C and shipped to St George's University of London for analysis.

### Cytokine production

Cytokine/chemokine protein was measured in supernatant fluids from PBMC cultures using R&D Fluorokine MAP kits (for MCP-2, GM-CSF, IL-2, IL-5, IL-10, IL-12, IFN-γ, and TGF-β) following the manufacturer's instructions and the plates were read on a BioRad Luminex reader. Samples were run in duplicate blind to infection group. Kits of the same lot were used and all assays were run on the same day. Sensitivities for these assays were 35–70 pg/mL and 1.6 pg/mL for IL-10.

### Microarray analyses

RNA extraction was performed using miRNeasy Mini Kit (Qiagen), co-purifying total RNA and microRNA. cDNA and cRNA synthesis and labelling was done using the Illumina TotalPrep 96 kit (Ambion, Austin, TX). Quantity and quality of the extracted RNA and cRNA was determined using Bioanalyzer (Agilent technologies, Palo Alto, CA) and Nanodrop ND1000 (Nanodrop Technologies, Wilmington, DE) and was comparably high between all subjects included in the analysis. Biotinylated cRNA (or microRNA) was hybridized to the arrays using Bead Station (version 3.1) blind to infection group. Illumina Human WG-6 v2 Beadchips (Illumina, San Diego, CA) were used to generate expression profiles of more than 48,000 transcripts. MicroRNA levels were measured using a microRNA expression panel (Illumina). After hybridization, chips were scanned on an Illumina BeadArray Reader and raw intensities were extracted using Illumina BeadStudio Gene Expression Module. Expression intensities were log base 2-transformed and quantile normalized using median expression intensity. 36798 probes out of 48701 were called present after background correction and normalization. Genespring software (Agilent) was used to perform quality control.

### Statistical analyses

Cytokine production was compared between the 3 infection groups and for two different infection effects: a STH infection effect (combined chronic and light vs. uninfected groups) and a chronic STH infection effect (chronic vs. combined light and uninfected groups). Non-parametric tests were used to compare cytokine and antibody levels between groups: Mann-Whitney for two-group and Kruskall-Wallis for three-group comparisons with the Bonferroni correction for multiple comparisons. Principal components analysis (PCA) is a widely used data reduction method used to reduce often highly correlated variables such as cytokines into a smaller number of uncorrelated variables called principal components. PCA was used to summarize multiple cytokine parameters to a small number of principal components as described elsewhere [10]. PCA was done unscaled on the logarithm of the values. Mean values of the first principal component were then compared using Student's t test between infection groups by bootstrap with 100,000 replicates [11]. Statistical analyses were done using STATA, version 10 (Statacorp, College Station, TX). Genespring software (Agilent) was used for analysis of microarray data. Comparisons were made for each of the STH infection and chronic STH infection effects. Because of the effects of averaging of gene expression across groups, the component comparisons for each infection effect were calculated. Differentially expressed (DE) genes were defined by a corrected P value<0.05 (adjusted for multiple test correction using the Benjamini and Hochberg method [12]) and a fold change (FC) cut off value of 1.5 for at least one of the comparisons. miRanda algorithm was used to scan miRNA sequences against 3′ UTR sequences on Illumina Human WG-6 chip searching for maximal local complementarity alignments and yielding scores that reflect the total miRNA vs. UTR alignment. Microarray and miRNA array data were uploaded onto the MIAME-based database ArrayExpress (accession numbers E-TABM-938 and E-TABM-939, respectively).

## Results

### Characteristics of study population

Details of the 60 children studied are provided in the Methods section and their baseline characteristics are summarized in Table 1. Frequencies of potential confounders were not significantly different between the groups with the exception of crowding (P = 0.001). All children in the chronic infection group and 65% of children in the light infection group were co-infected with *T. trichiura*. Levels of polyclonal and anti-*Ascaris* IgE increased across the groups from uninfected to chronic infection groups (Table 1).

**Table 1:** Baseline characteristics of the 3 infection groups.

Baseline Characteristics	Uninfected(n = 20)	Light(n = 20)	Chronic(n = 20)
Age, median years [range]	9 (7–12)	9 (7–12)	9 (7–12)
Sex (male/female)	7/13	9/11	10/10
**Socioeconomic factors**			
Monthly income, median US$ [range]	210 [50–1,000]	170 [30–800]	200 [50–400]
Latrine, %	90	95	69
Crowding, median [range]†	1.9 [0.8–6.0]	2.7 [1.0–6.0]	3.2 [1.7–10.0]
**Maternal educational level, %**			
Illiterate	15	47	69
Primary education	45	26	13
Secondary education	35	16	0
Don't know	5	11	18
Nutritional status			
Anemia, %	10	5	21
BMI, median [range]	16.9 [13.2–22.7]	16.4 [10.2–25.8]	15.0 [11.7–19.5]
**STH infection** *			
Any, %	0	100	100
*A. lumbricoides*, %	0	100	100
Infection intensity, median# epg [range]	0	552 [0–14,742]*	5,436 [0–46,368]*
*T. trichiura*, %	0	65	100
Infection intensity, median# epg [range]	0	0 [0–16,240]*	700 [0–4,445]*
**Antibodies**			
Total IgE, median IU/mL [range]	223 (60–975)	218 (40–5,000)	1,164 (141–5,000)
*Ascaris* IgE median kU/L [range]‡	0.35 (0.35–67.7)	0.83 (0.35–23.8)	7.00 (0.35–81.90)
*A. lumbricoides* IgG, median [range]	9 (2–19)	42 (8–57)	49 (14–481)
*A. lumbricoides* IgG4, median [range]	2 (2–8)	3 (2–14)	178 (39–1,000)

*The discrepancy between prevalence and median egg counts are because of differences in detection rates between the Kato-Katz and concentration methods.

# Median of mean egg counts.

**‡:** The range of detection of this assay is from 0.35–100 kU/L.

**†:** Crowding defined as persons per sleeping room. STH – soil-transmitted helminth.

### Cytokine production by PBMCs

**Figure 1:**
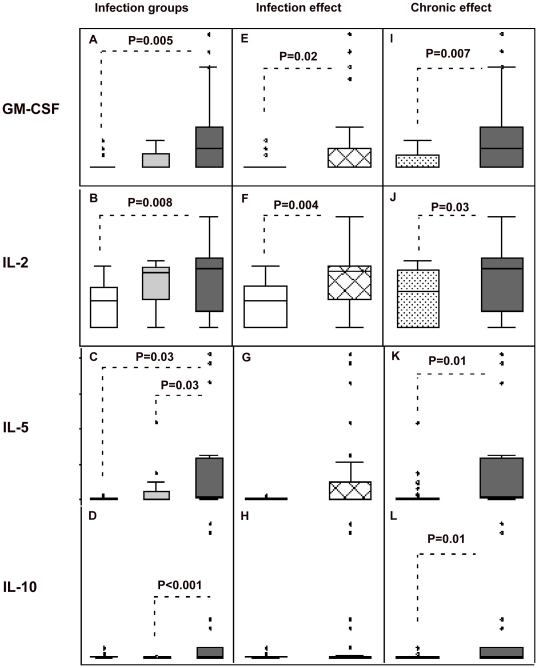
Production of cytokines, GM-CSF, IL-2, IL-5, and IL-10 by unstimulated peripheral blood mononuclear cells. Figures 1A–D: STH infection groups - uninfected (clear boxes), light (light grey), and chronic (dark grey) infections. Figures 1E–H: STH infection effect – uninfected (clear) and infected (hatched). Figures 1I–L: chronic STH infection effect: non-chronic [light infection group+uninfected]) (dotted) and chronic (dark grey) infections. Shown are box plots with median (central line), inter-quartile range (box margins), and 95% confidence intervals (bars). Cytokine concentrations are pg/mL. Only comparisons with P<0.05 are shown. P values in Figures 1A–D are corrected for multiple comparisons.

Comparisons between the three groups of production of cytokines and the chemokine MCP-2, by unstimulated PBMCs showed significant heterogeneity of effect for GM-CSF (P = 0.01), IL-2 (P = 0.01), IL-5 (P = 0.04), and IL-10 (P = 0.0007) (Figure 1A–D). No significant differences were observed between the three groups for the other cytokines and MCP-2: MCP-2 (uninfected, median 170 pg/mL, interquartile range [IQR] 158-188; light, median 165, IQR 70-199; chronic, median 170, IQR 156-363), IL-12 (uninfected, median 35 pg/mL, IQR 35-35; light, median 35, IQR 35-35; chronic, median 35, IQR 35-148), IFN-γ (uninfected, median 70 pg/mL, IQR 70-155; light, median 70, IQR 70-143; chronic, median 70, IQR 70-382), and TGF-β (uninfected, median 265 pg/mL, IQR 160-465; light, median 285, IQR 235-427; chronic, median 241, IQR 201-427). Post-hoc comparisons showed significantly elevated production of GM-CSF (P = 0.005), IL-2 (P = 0.008), and IL-5 (P = 0.03) in the chronic infection compared to uninfected groups and elevated production of IL-5 (P = 0.03) and IL-10 (P = 0.0008) in the chronic versus light infection groups (Figures 1A–D). The STH infection effect (combined light and chronic infection groups) was associated with greater production of GM-CSF (P = 0.02) and IL-2 (P = 0.004) (Figures 1E & F) and the chronic STH infection effect with greater levels of GM-CSF (P = 0.007), IL-2 (P = 0.03), IL-5 (P = 0.01), and IL-10 (P = 0.01) (Figures 1 I–L). PCA yielded a single component that accounted for 70% of total variation with coefficients of −0.119 for MCP-2, −0.950 for IL-5, and −0.272 for IL-10 (i.e. a component representing an immune response dominated by IL-5 and IL-10). All other cytokine loadings for this component were small (i.e. <0.1 or >−0.1). The remaining components, that each accounted for less than 20% of variance, were not analysed further. Comparisons between mean values of PC1 for infection groupings showed significant differences for the chronic (P = 0.004) and STH infection (P = 0.007) effects. Means for PC1 were different between chronic and light groups (P = 0.02) or uninfected groups (P = 0.001).

### Gene expression profiles

**Figure 2:**
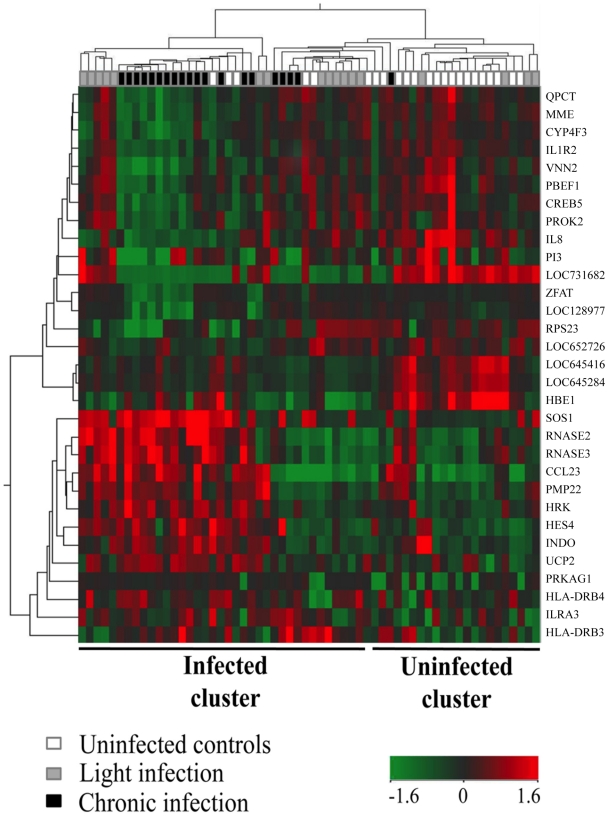
Heat map representing hierarchical clustering of gene expression for the 60 study children. Gene expression profiles are shown for uninfected children, and those with light and chronic infections. Red indicates increased gene expression and green decreased gene expression.

The gene expression profiles in peripheral blood of the three different infection groups were analysed using enriched RNA on microarrays covering 48,000 human transcripts. Hierarchical cluster analysis showed clustering of gene expression by infection group – there was evidence of differences between the three groups in terms of their gene expression profiles with the light infection group representing a transitional profile between uninfected and chronic infection groups (Figure 2). Overall relatively few genes were significantly differentially expressed for the STH infection (uninfected vs. light/chronic infection groups) and chronic STH infection (chronic vs. light/uninfected groups) effects after controlling for multiple comparisons. Findings with differential expression of at least 1.5-fold change (FC) and corrected P value of ≤0.05 are shown in Table 2. The corrected P value for each of the two comparisons (STH infection effect - combined light and chronic infection vs. uninfected groups; chronic STH infection effect - chronic vs. combined light and uninfected groups) and FCs for each of the component comparisons (STH infection effect – light vs. uninfected and chronic vs. uninfected; chronic STH infection effect: chronic vs. uninfected and chronic vs. light) are shown. Differentially regulated genes were: CCL23 (chemokine ligand 23), CREB5 (cAMP responsive element binding protein 5), CYP4F3 (cytochrome P450 polypeptide), HBE1 (hemoglobin, epsilon 1), HES4 (hairy and enhancer of split 4), HLA-DRB3 (MHC class II, DR beta 3), HLA-DRB4 (MHC class II, DR beta 4), HRK (Harakiri, BCL interacting protein), IL-1R2 (interleukin-1 receptor type II), IL-8 (interleukin-8), IDO (indoleamine 2,3-dioxygenase), LILRA3 (leukocyte immunoglobulin-like receptor, subfamily A member 3), LOC128977 (chromosome 22 open reading frame 39), LOC645284 (hypothetical protein LOC645284), LOC652726 (similar to ankyrin repeat domain 36), LOC731682 (HLA class II, DQ(1) alpha chain precursor-like), MME (membrane metalloendopeptidase), PBEF1/NAMPT (nicotinamide phosphoribosyltransferase), PI3 (peptidase inhibitor 3), PMP22 (peripheral myelin protein), PRKAG1 (protein kinase, AMP activated, gamma 1), PROK2 (prokineticin 2), QPCT (glutamyl-peptide cyclotransferase), RNASE2 (eosinophil derived neurotoxin), RNASE3 (eosinophil cationic protein), RPS23 (ribosome protein S23), SHANK2 (SHANK2 SH3 and multiple ankyrin repeat domains 2), SOS1 (son of sevenless homolog 1), UCP2 (uncoupling protein 2), VNN2 (vanin 2), and ZFAT1 (zinc finger and AT hook domain containing). Three genes were differentially regulated for the chronic but not the STH infection effects (up-regulated - HLA-DRB3, LILRA3; down-regulated - ZFAT1). Within the chronic STH infection effect, there was evidence of relatively greater effects on gene expression of chronic than light infection groups when compared to the uninfected groups. For example, in the chronic vs. uninfected group comparison there was a 2.1-fold up-regulation of CCL23 expression, while expression was upregulated 1.6-fold in light vs. uninfected and 1.3-fold in chronic vs. light groups. Overall, the chronic infection effect was associated with genes regulating: 1) inflammation: ZFAT1 [down regulated, anti-apoptotic regulator], IL-1R2 [down, inducer of cell migration]; LILRA3 [up-regulated, potential anti-inflammatory molecule]; UCP2 [up-regulated, negative regulator of reactive oxygen species production by macrophages]; 2) neutrophil-mediated inflammation: IL-8 [down, neutrophil chemotaxis and activation], VNN2 [down, transendothelial migration of neutrophils], PBEF1 [down, inhibition of neutrophil apoptosis], and CCL23 (up, inhibition of production by and release of neutrophils from the bone marrow]; 3) antibacterial immunity (down, skin antimicrobial peptide PI3); 4) immune homeostasis, a) the induction of homeostatic mechanisms during infection (up, IDO), b) chemotaxis of resting T cells and monocytes (up, CCL23), and c) activation of cell apoptosis (up, HRK); and 5) the capacity of eosinophils to kill helminth parasites (up, RNASE2 and RNASE3). CYP4F3, an inactivator of inflammatory mediator leukotriene B4, was downregulated and MHC class II antigen gene expression (HLA-DRB3 and HLA-DRB4) were upregulated.

**Table 2:** Differential expression of genes for STH infection and chronic STH infection effects.

Gene identifier	STH infection effect*			Chronic STH infection effect**		
	Light vs. Uninfected	Chronic vs. Uninfected	*Corrected P value	Chronic vs. Light	Chronic vs. Uninfected	*Corrected P value
CCL23	1.62	2.086	0.008	1.283	2.086	0.008
CREB5	1	−1.518	0.003	−1.518	−1.518	0.003
CYP4F3	−1.117	−1.543	0.003	−1.382	−1.543	0.003
HBE1	−2.587	−2.007	2.76E-04	1.289	−2.007	2.71E-04
HES4	1.366	1.775	0.012	1.299	1.775	0.012
HLA-DRB3				1.601	1.265	0.033
HLA-DRB4	1.368	1.51	0.033	1.104	1.51	0.034
HRK	1.363	1.586	0.005	1.163	1.586	0.005
IL1R2	−1.19	−1.557	3.90E-04	−1.309	−1.557	3.91E-04
IL8	−1.357	−2.014	1.92E-04	−1.485	−2.014	1.84E-04
IDO	1.205	1.521	0.029	1.262	1.521	0.031
LILRA3				1.506	1.391	0.044
LOC128977	1.053	−1.562	3.21E-05	−1.645	−1.562	2.92E-05
LOC645284	−1.385	−1.518	2.76E-04	−1.096	−1.518	2.71E-04
LOC652726	−1.326	−1.524	1.92E-04	−1.149	−1.524	1.84E-04
LOC731682	−1.173	−2.394	0.002	−2.041	−2.394	0.002
MME	−1.136	−1.593	0.0012	−1.402	−1.593	0.001
PBEF1/NAMPT	−1.151	−1.537	0.008	−1.335	−1.537	0.008
PI3	1.21	−1.698	0.011	−2.054	−1.698	0.012
PMP22	1.501	1.542	0.011	1.028	1.542	0.011
PRKAG1	1.834	1.858	1.21E-07	1.013	1.858	1.33E-07
PROK2	1.005	−1.523	0.005	−1.531	−1.523	0.005
QPCT	−1.219	−1.539	0.006	−1.263	−1.539	0.006
RNASE2	1.433	2.119	0.008	1.479	2.119	0.009
RNASE3	1.1963	1.796	0.028	1.501	1.796	0.029
RPS23	−1.1873	−1.622	0.041	−1.366	−1.622	0.043
SHANK2	−1.483	−1.555	5.08E-04	−1.049	−1.555	5.12E-04
SOS1	1.283	2.141	3.08E-04	1.669	2.141	3.06E-04
UCP2	1.179	1.512	0.012	1.282	1.512	0.013
VNN2	−1.055	−1.578	0.01	−1.496	−1.578	0.011
ZFAT1				−1.513	−1.473	2.03E-06

*STH infection effects represent a comparison of infected (chronic+light) vs. uninfected groups.

**Chronic STH infection effects represent a comparison of chronic vs. non-chronic (light+uninfected groups) groups. P values are corrected for multiple comparisons and represent the overall STH infection and chronic infection effects.

The purified RNA populations were also analysed on a specific microarray for monitoring miRNA expression profiles. Both infection effects were associated with the significant differential expression of a single microRNA, hsa-let-7d (STH infection effect, FC: −20.7, corrected P = 0.012; chronic STH effect, −21.3, corrected P = 0.010). Data for differential expression of microRNAs for both infection effects with −1.5<FC>1.5 and uncorrected P values of ≤0.05 are provided in the online archive (Table S1). We searched for putative targets for hsa-let-7d using the miRBase targets database to see if it could be responsible for the regulation of either the differentially expressed genes or cytokine production observed during chronic infection. Because miRNA would be expected to be inversely associated with such targets, we looked for putative gene targets that had expression profile opposite to this microRNA. Hsa-let-7d was inversely associated with CCL23 (score 17.2, P base = 0.03). Results of this analysis for the whole data set are provided in the online archive (Table S2). Of the 4 cytokines whose production was increased during chronic infection, IL-2 is a putative target for hsa-let-7d.

## Discussion

Chronic STH infections of humans have been associated with effects on immune responses to vaccines [3], [4] and the modulation of inflammatory reactions in the skin [13], [14], lungs [15], and intestine [6]. A mechanism by which STH infections may have wide-ranging effects on host immunity is through alterations of immune homeostasis. In the present study, we investigated the effects of chronic STH infections on immune homeostasis by measurement of the accumulation of cytokines *in vitro* by unstimulated PBMCs and gene expression by unstimulated whole blood.

Our data show that PBMCs from children with chronic infections produce higher levels of GM-CSF, IL-2, IL-5, and IL-10 spontaneously compared to those from children without chronic infections. GM-CSF and IL-5 have important roles in eosinophil recruitment, activation and survival in the tissues [16]. Elevated levels of GM-CSF and IL-5 in plasma from individuals infected with helminths have been associated with enhanced eosinophil survival [16]. Given that chronic infection effect was associated also with an upregulation of the genes for eosinophil-derived neurotoxin (RNASE2) and eosinophil cationic protein (RNASE3), such observations may indicate a mechanism by which parasite numbers are controlled during chronic infections. In addition, IL-2 appears to be important for the induction of IL-5 in PBMCs in helminth-infected individuals [17]. Previous studies have shown that the frequency of individuals producing detectable IL-10 spontaneously is greater among children living in an unhygienic environment [18] and among those with STH infections [19], and IL-10 production has been associated with immune regulation during chronic helminth infections [20]–[22]. The elevated production of IL-10 spontaneously during helminth infections could contribute to immune homeostasis by raising the threshold for the induction of effector inflammatory responses in the tissues [23]. Data reduction provided evidence that chronic infections were associated with a cytokine profile (IL-5 and IL-10), consistent with a modified Th2 immune response. The modified Th2 response was originally described to explain a ‘tolerant immune response’ to cat allergen that develops on high levels of allergen exposure and is associated with elevated allergen-specific IgG4 and T cells expressing IL-5 and IL-10 [24]. The modified Th2 response thus describes a tolerant phenotype that is distinct from classical IL-4-induced Th2 immunity that is considered to drive allergic inflammation. Subsequently, this term has been used to describe a similar immune response characteristic of chronic experimental helminth infections in mouse models [25]. Our data indicate that a modified Th2-like response develops among children with chronic STH infections. Such an immune response representing a regulated Th2 response may be an important feature of balanced parasitism that ensures parasite survival but protects the host from Th2-induced pathology.

Several genes were differentially regulated during chronic STH infection in this study. Given that we analysed unstimulated peripheral blood collected during chronic infections, we expected to see subtle alterations in the gene expression profiles using a cut-off of at least a 1.5-fold change in gene expression. Large perturbations in gene expression might be expected to have significant morbid consequences and would be not be expected to occur in the context of a state of balanced parasitism as is the case of chronic STH infections in humans. The STH and chronic STH effects were associated with the differential regulation of genes linked with neutrophil-mediated inflammation: down-regulation of genes linked with the chemotaxis (IL-8) [26], activation (IL-8) [26], transendothelial migration (VNN2) [27], and inhibition of apoptosis (PBEF1) [28] of neutrophils, while a gene that inhibits the production by and release of neutrophils from the bone marrow (CCL23) [29] was up-regulated. Such gene regulation could have the effect of modulating potential pathology-inducing responses to migratory larvae of *A. lumbricoides* – neutrophils play a prominent role in pathology caused by the migration of *Ascaris* larvae through the lungs [30] and other tissues [31]. Both infection effects were associated also with the up-regulation of eosinophil mediators with potent helminthotoxic functions (RNASE2 [eosinophil-derived neurotoxin] and RNASE3 [eosinophil cationic protein] [32] perhaps indicating an up-regulation of signals promoting eosinophilia (i.e. in conjunction with the elevated production by PBMCs of spontaneous GM-CSF and IL-5 observed in chronic infections) and helminth-killing ability. In addition, we observed up-regulation of several genes considered to be involved in immune homeostasis (e.g. IDO [33], CCL23 [34], and HRK [35]). Immune homeostasis reflects a complex state in which interactions between innate and adaptive immunity prevent inappropriate inflammatory responses to harmless immunogens but permit the development of appropriate but controlled immune responses to harmful exogenous immunogens. IDO (indoleamine 2,3-dioxygenase) has a key role in the suppression of acute inflammatory responses and the promotion of immune tolerance [33] and has been shown to contribute to the establishment of chronic fungal infections by induction of regulatory T cells and the suppression of the Th17-inflammatory pathway [36]. IDO may have also a role in the modulation of allergic inflammatory responses [33] and up-regulation of IDO as observed in the present study may, therefore, be relevant to the development of chronicity during STH infections. The chronic STH effect was associated also with the differential regulation of genes for HLA class II molecules: HLA-DRB3 and HLA-DRB4 were upregulated but LOC731682 (HLA class II, DQ(1) alpha chain precursor-like) was downregulated. The biological significance of these observations are not clear but could be addressed in future studies.

There are limited data on the potential regulatory role of miRNAs during infectious diseases – mice deficient for miR-155 were unable to develop immunity to *Salmonella typhimurium* after immunization [37], cholangiocytes infected with *Cryptosporidium parvum* showed impaired expression of let-7i [38], and miR-155 has been suggested to have a role in the negative regulation of inflammation associated with *Helicobacter pylori* infection [39]. To our knowledge, this is the first reported study to investigate the potential role of miRNAs during chronic helminth infections. In our analysis we observed the significant differential expression of a single miRNA, hsa-let-7d, that was down-regulated approximately 20-fold for both STH and chronic infection effects suggestive of the progressive down-regulation of this gene from uninfected to infected to chronically infected children. There is a growing literature on the potential role of hsa-let-7d in disease processes: decreased expression has been associated with decreased survival in head and neck squamous cell carcinoma [40] and with impairment of pulmonary function and development of disease in a murine model of idiopathic pulmonary fibrosis [41]. Expression of hsa-let-7d may have a role also in iron metabolism in erythroid cells and the intestine [42]. CCL23 was found to be a putative target for hsa-let-7d. The strong down regulation of hsa-let-7d during STH infection was associated with an increased expression of CCL23, a gene with several important functions including the suppression of the production and release of neutrophils from the bone marrow [29] and of colony formation by immune cells *in vitro* [43]. There is evidence from murine models that antagonism of specific miRNAs may attenuate the inflammatory and clinical processes associated with Th2-driven airways inflammation [44]. The specific role of different miRNAs in modulating immune cell function is only now starting to be unravelled, but our data provide some support for a potential role of hsa-let-7d in modulating the immune response during chronic STH infection.

### Study limitations

An important limitation was the small sample size that limited the power to detect relatively small changes in the expression of a large number of genes in peripheral blood. The analysis was exploratory and our data provide evidence for the differential expression of several potentially relevant genes such as IL-8 and the microRNA hsa-let-7d. The sample size was limited by the high cost of these analyses. We had to screen a relatively large population of school children to select our study population using strict selection criteria based on the presence or absence of STH infection in serial stool samples and anti-*Ascaris* IgG and IgG4 in plasma samples. Although this selection process allowed us to identify well-defined sub-groups of children that were of specific interest for our study objectives, our findings cannot necessarily be generalized to all children with STH infections, particularly as the prevalence of our STH infection sub-groups as defined will be extremely variable between populations. We defined chronic infection on the basis of repeated stool samples being positive for *A. lumbricoides* and the presence of anti-*A. lumbricoides* IgG4 antibodies. The presence of parasite-specific IgG4 is a well-recognised feature of chronic helminth infections [45] including those with STH parasites [19], [46]. The use of anti-*A. lumbricoides* IgG4 to define chronic STH infection could have biased the results with respect to IL-10 production because this cytokine differentially induces IgG4 in B cells [47]. However, elevated spontaneous IL-10 has been reported in children with STH infections previously [19], [48], [49]. We defined homeostasis by the measurement of basal or unstimulated cytokine production by PBMCs and gene expression in unstimulated whole blood [18], [50]. Such a definition may be subject to significant limitations: 1) Measurements were taken at a single time point. Prospective studies conducted from the time of first exposures to STH infections in infancy will be required to indicate more strongly a causal association between the development of chronic STH infection and such effects on immune responses. 2) The measurement of the accumulation of cytokines over 5 days by unstimulated PBMCs may be complicated by other processes such as the differentiation of monocytes into macrophages and cell death. Such *in vitro* artefacts would be expected to occur in all cultures irrespective of study group and would mask rather than emphasize inter-group differences. Further, our findings are consistent with those of previous studies of helminth-infected individuals in which unstimulated whole blood or PBMCs were cultured for variable periods of one to five days [18], [19], [22], [48], [49], [51], [52]. Recently the cells producing IL-10 spontaneously in helminth-infected individuals have been identified as CD4+ T regulatory type 1 cells [50]. Further, we observed also elevated production of IL-10 (P = 0.04) and a trend of increased IL-5 in chronic versus non-chronic infections in PBMC cultures stimulated with *A. lumbricoides* antigen (data not shown), an observation that is consistent with the findings from unstimulated cultures. Because the study was cross-sectional and we had no data on previous parasite infections, we cannot exclude heavy previous infections in the light infection group or past infections in the uninfected group although, in the case of the latter, the children were unlikely to have been infected recently because of the absence of *A. lumbricoides*-specific IgG antibodies. However, such misclassification would be expected to reduce the size of inter-group differences. The comparisons were not controlled for potential confounders and there were differences, albeit non-significant, between the groups in nutritional factors such as the prevalence of anemia. Other unmeasured confounding factors may have differed between the groups. Important co-infections with powerful effects on the immune response such as HIV, malaria, and tuberculosis were of very low prevalence in our study population. However, we cannot exclude confounding as an alternative explanation for our study findings.

### Conclusion

The present study has provided evidence that PBMCs of children with chronic STH infections produce elevated levels spontaneously GM-CSF, IL-2, IL-5, and IL-10, the latter two cytokines being suggestive of a modified Th2 response. We were able to detect the differential regulation of several genes during STH infection including genes suggestive of the suppression of neutrophil-mediated inflammation and perhaps also the up-regulation of immune homeostasis. Both STH and chronic STH infection effects were associated with the down-regulation of microRNA has-let-7d possibly indicating a role for this gene during infection. The data from the present study underlie the complexity of the molecular processes associated with the development of chronic STH infections. A better understanding of the mechanisms by which STHs modulate anti-parasite inflammatory responses may lead to the development of new therapies for inflammation.

### Gene accession numbers

CCL23 (NM_145898.1), CREB5 (NM_182898.2), CYP4F3 (NM_000896.1), HBE1 (NM_005330.3), HES4 (NM_021170.2), HLA-DRB3 (NM_022555.3), HLA-DRB4 (NM_021983.4), HRK (NM_003806.1), IL1R2 (NM_173343.1), IL-8 (NM_000584.2), IDO (NM_002164.3), LILRA3 (NM_006865.2), LOC128977 (NM_173793.3), LOC645284 (XM_932788.1), LOC645416 (XM_928457.1), LOC652726 (XM_942351.2), LOC731682 (XM_001129369.1), MME (NM_000902.3), PBEF1/NAMPT (NM_005746.2), PI3 (NM_002638.2), PMP22 (NM_153321.1), PRKAG1 (NM_002733.3), PROK2 (NM_021935.2), QPCT (NM_012413.3), RNASE2 (NM_002934.2), RNASE3 (NM_002935.2), RPS23 (NM_001025.4), SOS1 (NM_005633.2), UCP2 (NM_003355.2), VNN2 (NM_004665.2), and ZFAT1 (NM_001029939.1).

## Supporting Information

Table S1 **The cut-off for inclusion was fold difference in expression ≥1.5 and uncorrected P≤0.05.**
(DOC)

Table S2 **Shown are scores and P-bases.**
(DOC)

Text S1 STROBE checklist (DOC)
