# Supplementary material for: Effects of Chronic Ascariasis and Trichuriasis on Cytokine Production and Gene Expression in Human Blood: A Cross-Sectional Study
Source: PLoS Negl Trop Dis. 2011 Jun 7;5(6):e1157. doi: 10.1371/journal.pntd.0001157 (PMC3110165; doi:10.1371/journal.pntd.0001157)
Supplement: Table S1 — The cut-off for inclusion was fold difference in expression ≥1.5 and uncorrected P≤0.05. (DOC) [file pntd.0001157.s001.doc]

Table S1. Differential expression of miRNAs for STH infection (infected vs. uninfected) and chronic STH infection (chronic vs. non-chronic [light infection group+ uninfected]) effects.

| miRNA | Infection effect | | Chronic infection effect | |
| --- | --- | --- | --- | --- |
|  | Fold change | P value | Fold change | P value |
| hsa-let-7d | -20.7 | 0.00002 | -21.3 | 0.00002 |
| hsa-miR-18a |  |  | -4.4 | 0.01 |
| hsa-miR-29b | -5.1 | 0.04 | -5.1 | 0.04 |
| hsa-miR-29c | -2.8 | 0.03 | -2.8 | 0.03 |
| hsa-miR-30a-5p | -7.7 | 0.003 | -7.7 | 0.003 |
| hsa-miR-103 |  |  | -6.1 | 0.02 |
| hsa-miR-132 |  |  | -2.1 | 0.04 |
| hsa-miR-142-3p |  |  | -2.9 | 0.03 |
| hsa-miR-197 | -3.1 | 0.04 | -3.1 | 0.04 |
| hsa-miR-185 | -2.9 | 0.04 | -3.1 | 0.04 |
| hsa-miR-200c | -2.3 | 0.004 | -2.3 | 0.004 |
| hsa-miR-196b |  |  | -1.7 | 0.03 |
| hsa-miR-296 |  |  | -2 | 0.05 |
| hsa-miR-324-3p | -6.2 | 0.009 | -5.5 | 0.02 |
| hsa-miR-346 | -5 | 0.05 | -5 | 0.05 |
| hsa-miR-422b | -1.8 | 0.02 | -1.8 | 0.02 |
| hsa-miR-454-3p | -2 | 0.004 | -1.9 | 0.01 |
| hsa-miR-454-5p | -2 | 0.04 | -2 | 0.04 |
| hsa-miR-570 |  |  | -2.4 | 0.03 |
| hsa-miR-574 | -1.6 | 0.04 | -1.6 | 0.04 |
| hsa-miR-618 | -1.7 | 0.02 | -1.7 | 0.02 |
| hsa-miR-625 | -2 | 0.04 | 2.7 | 0.005 |
| HS_24 | 2.6 | 0.008 | -2.5 | 0.002 |
| HS_98 | -1.7 | 0.05 | -1.7 | 0.05 |
| HS_192.1 | 2.5 | 0.05 | 2.5 | 0.05 |
| HS_261.1 |  |  | -18.2 | 0.01 |
| HS_263.1 |  |  | -1.6 | 0.04 |
