# Supplementary material for: Effects of Chronic Ascariasis and Trichuriasis on Cytokine Production and Gene Expression in Human Blood: A Cross-Sectional Study
Source: PLoS Negl Trop Dis. 2011 Jun 7;5(6):e1157. doi: 10.1371/journal.pntd.0001157 (PMC3110165; doi:10.1371/journal.pntd.0001157)
Supplement: Table S2 — Shown are scores and P-bases. (DOC) [file pntd.0001157.s002.doc]

Table S2. Differentially expressed miRNAs that were inversely associated

with the differentially expressed genes.

| **Gene target** | **microRNA** | **Score** | **P-base** |
| --- | --- | --- | --- |
| CCL23 | hsa-let-7c, hsa-let-7d | 16.8; 17.2 | 0.04; 0.03 |
| HBE1 | mir-346, mir-197 | 18.0, 16.2 | 0.006; 0.05 |
| HRK | mir-570 | 16.6 | 0.06 |
| IDO | HS_32 | 17.2 | 0.02 |
| PMP22 | mir-29b, mir-29c | 17.8; 17.0 | 0.01; 0.02 |
| PRKAG1 | mir-574 | 16.8 | 0 |
| RNASE2 | mir-185 | 17.1 | 0.04 |
| SOS1 | mir-181a | 15.4 | 0.09 |
| TFF3 | mir-497 | 16.4 | 0.02 |
| PI3 | mir-675 | 17.4 | 0.01 |
| QPCT | mir-338-5p | 16.9 | 0.05 |
