# Supplementary material for: Effects of Chronic Ascariasis and Trichuriasis on Cytokine Production and Gene Expression in Human Blood: A Cross-Sectional Study
Source: PLoS Negl Trop Dis. 2011 Jun 7;5(6):e1157. doi: 10.1371/journal.pntd.0001157 (PMC3110165; doi:10.1371/journal.pntd.0001157)
Supplement: Text S1 STROBE checklist — (DOC) [file pntd.0001157.s003.doc]

STROBE Statement—Checklist of items that should be included in reports of ***cross-sectional studies***

|  | Item No | Recommendation |
| --- | --- | --- |
| **Title and abstract** | 1 | (*a*) Indicate the study’s design with a commonly used term in the title or the abstract – see lines 1-3 |
|  |  | (*b*) Provide in the abstract an informative and balanced summary of what was done and what was found – see pages 2-3 |
| Introduction | | |
| Background/rationale | 2 | Explain the scientific background and rationale for the investigation being reported: see pages 5-6 |
| Objectives | 3 | State specific objectives, including any prespecified hypotheses: lines 96-99 |
| Methods | | |
| Study design | 4 | Present key elements of study design early in the paper: see line 102 |
| Setting | 5 | Describe the setting, locations, and relevant dates, including periods of recruitment, exposure, follow-up, and data collection: see pages 7-8 |
| Participants | 6 | (*a*) Give the eligibility criteria, and the sources and methods of selection of participants: see pages 7-8 |
| Variables | 7 | Clearly define all outcomes, exposures, predictors, potential confounders, and effect modifiers. Give diagnostic criteria, if applicable: see pages 7-8; data on confounders is provided in Table 1. |
| Data sources/ measurement | 8* | For each variable of interest, give sources of data and details of methods of assessment (measurement). Describe comparability of assessment methods if there is more than one group: see pages 8-10 |
| Bias | 9 | Describe any efforts to address potential sources of bias: |
| Study size | 10 | Explain how the study size was arrived at: see lines 163-164 & 175 |
| Quantitative variables | 11 | Explain how quantitative variables were handled in the analyses. If applicable, describe which groupings were chosen and why: see lines 111-121 |
| Statistical methods | 12 | (*a*) Describe all statistical methods, including those used to control for confounding: see pages 10-11 |
|  |  | (*b*) Describe any methods used to examine subgroups and interactions: see lines 190-192; 205-207 |
|  |  | (*c*) Explain how missing data were addressed: not applicable |
|  |  | (*d*) If applicable, describe analytical methods taking account of sampling strategy: not applicable |
|  |  | (*e*) Describe any sensitivity analyses: not applicable |
| Results | | |
| Participants | 13* | (a) Report numbers of individuals at each stage of study—eg numbers potentially eligible, examined for eligibility, confirmed eligible, included in the study, completing follow-up, and analysed: lines 108-135 |
|  |  | (b) Give reasons for non-participation at each stage; see lines 108-135 |
|  |  | (c) Consider use of a flow diagram |
| Descriptive data | 14* | (a) Give characteristics of study participants (eg demographic, clinical, social) and information on exposures and potential confounders: see lines 217-219; Table 1 |
|  |  | (b) Indicate number of participants with missing data for each variable of interest: no missing data for 60 subjects analysed. |
| Outcome data | 15* | Report numbers of outcome events or summary measures: pages 12-16 |
| Main results | 16 | (*a*) Give unadjusted estimates and, if applicable, confounder-adjusted estimates and their precision (eg, 95% confidence interval). Make clear which confounders were adjusted for and why they were included: see lines 478-484 |
|  |  | (*b*) Report category boundaries when continuous variables were categorized: not applicable for study outcomes |
|  |  | (*c*) If relevant, consider translating estimates of relative risk into absolute risk for a meaningful time period: not relevant |
| Other analyses | 17 | Report other analyses done—eg analyses of subgroups and interactions, and sensitivity analyses: not done |
| Discussion | | |
| Key results | 18 | Summarise key results with reference to study objectives: lines 487-498 |
| Limitations | 19 | Discuss limitations of the study, taking into account sources of potential bias or imprecision. Discuss both direction and magnitude of any potential bias: lines 433-484 |
| Interpretation | 20 | Give a cautious overall interpretation of results considering objectives, limitations, multiplicity of analyses, results from similar studies, and other relevant evidence: lines 487-498 |
| Generalisability | 21 | Discuss the generalisability (external validity) of the study results: lines 438-445 |
| Other information | | |
| Funding | 22 | Give the source of funding and the role of the funders for the present study and, if applicable, for the original study on which the present article is based: see Financial Disclosure Section. |

*Give information separately for exposed and unexposed groups.

**Note:** An Explanation and Elaboration article discusses each checklist item and gives methodological background and published examples of transparent reporting. The STROBE checklist is best used in conjunction with this article (freely available on the Web sites of PLoS Medicine at http://www.plosmedicine.org/, Annals of Internal Medicine at http://www.annals.org/, and Epidemiology at http://www.epidem.com/). Information on the STROBE Initiative is available at www.strobe-statement.org.
